# Molecular pathways linking chronic psychological stress to accelerated aging: mechanisms and interventions

**DOI:** 10.3389/fragi.2026.1743142

**Published:** 2026-02-04

**Authors:** Othman A. Alhazzaa, Hanin M. Abahussin, Majed A. Majrashi, Maryam S. Alotaibi, Mohammad N. Alkhrayef, Ziyad A. Alhamdan, Abdullah O. Alawad

**Affiliations:** 1 Healthy Aging Research Institute, Health Sector, King Abdulaziz City for Science and Technology, Riyadh, Saudi Arabia; 2 Bioengineering Research Institute, Health Sector, King Abdulaziz City for Science and Technology, Riyadh, Saudi Arabia; 3 Disability Research Institute, Health Sector, King Abdulaziz City for Science and Technology, Riyadh, Saudi Arabia

**Keywords:** aging, epigenetic, inflammation, mitochondria, psychological stress, stress response, telomere

## Abstract

Chronic psychological stress refers to repeated or prolonged exposure to adverse social or emotional threats that exceed an individual’s adaptive capacity. It is recognized as a risk factor for aging-associated diseases. A growing body of research has shown that there is a link between chronic psychological stress and accelerated aging. Here, we highlight recent findings on the interconnected relationship between chronic psychological stress and major aging hallmarks, including mitochondrial dysfunction, telomere attrition, cellular senescence, epigenetic alterations, inflammation, and genomic instability. We discuss the mechanisms by which chronic psychological stress may drive this effect and explore intervention strategies that could mitigate its adverse effects and promote healthy aging. Moreover, we address current research gaps and propose future research directions to improve our understanding of the intricate relationship between psychological stress and aging.

## 1 Introduction

Psychological stress refers to exposure to adverse social or emotional threats, including low socioeconomic status, financial strain, early life adversity, discrimination, work-related stress, and loneliness (Crimmins, 2020; Rentscher et al., 2020). Depending on the nature, duration, and intensity of the stress stimuli (i.e., the stressor), stress is commonly classified as either acute or chronic. Acute stress involves short-term exposure to a stressor and typically induces an adaptive, time-limited response. In contrast, chronic stress results from prolonged or repeated exposure to stressors. In today’s fast-paced and demanding society, individuals are experiencing higher levels of psychological stress than ever before (Daly and Macchia, 2023). Data from 149 countries show a continuous rise in daily emotional stress, increasing from 26% in 2007 to 38% in 2020 (Piao et al., 2024). In Saudi Arabia, the National Mental Health Survey of more than 4,000 participants aged 15–65 years old showed that 34% met the criteria for a mental health disorder at some point in their lives (AlTwaijri et al., 2019). Other studies have reported alarming findings on stress prevalence. For example, work-related psychological stress among healthcare professionals in Saudi Arabia has been reported to range from 56% to 83% (Alharbi et al., 2024; Almutairi et al., 2024; Alqarni et al., 2022). A meta-analysis of 25,814 male and female participants reported a 37% prevalence of depression among Saudi adults (Nour et al., 2023).

Exposure to chronic stress takes a significant toll on health and increases the risk of mortality (Keller et al., 2012; Prior et al., 2016). Psychological conditions such as depression and anxiety are associated with early onset and increased risk of age-related diseases, including dementia (Richmond-Rakerd et al., 2022; Sulkava et al., 2022), cardiovascular diseases (Krittanawong et al., 2023; Sara et al., 2023; Zeng et al., 2025), and diabetes (Cai et al., 2024). Several studies have demonstrated a link between chronic psychological stress and accelerated aging (Galkin et al., 2022; Wang et al., 2024). These findings support the concept that the association between chronic psychological stress and adverse health outcomes is driven, at least in part, by mechanisms that drive the aging process.

Aging is defined as the gradual deterioration of normal physiological and cellular function over time. The accumulation of impaired cells can lead to a loss of tissue function, ultimately resulting in the inability to maintain normal function and sustain life (Luo et al., 2020). Disruption of molecular and cellular functions during aging contributes to the onset of aging-associated diseases (Li et al., 2021). Over the years, a set of twelve aging hallmarks has been identified, including genomic instability, telomere shortening, epigenetic changes, loss of proteostasis, impaired macroautophagy, cellular senescence, mitochondrial dysfunction, deregulated nutrient sensing, stem cell exhaustion, altered intercellular communication, chronic inflammation, and microbiota dysbiosis (López-Otín et al., 2013; López-Otín et al., 2023).

In this review, we discuss recent findings on the impact of chronic psychological stress on aging and the molecular mechanisms that mediate this relationship. While all hallmarks contribute to the complex biology of aging, this review focuses on six that are most strongly supported by current evidence linking them to chronic psychological stress: cellular senescence, telomere attrition, epigenetic alterations, mitochondrial dysfunction, inflammation, and DNA damage. Then we propose potential intervention strategies to mitigate the adverse impacts of chronic psychological stress. Lastly, we highlight current gaps and propose directions for future research.

## 2 Physiological stress response pathways

**FIGURE 1:**
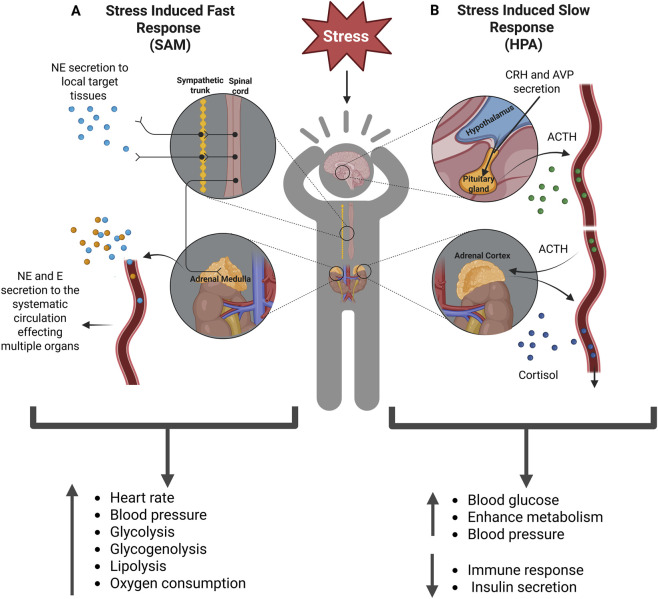
Schematic illustration of the stress response via the SAM and HPA axes. **(A)** Stress-induced fast response: stress activates the SAM, consequently resulting in the secretion of norepinephrine from postganglionic nerves. It induces the secretion of norepinephrine and epinephrine from the adrenal medulla into the systemic circulation. **(B)** Stress-induced slow response: When stress is perceived, CRH and AVP are released in the hypothalamus into the hypophysial portal system. This triggers the anterior pituitary to secrete ACTH. In turn, ACTH stimulates the adrenal cortex to produce and release cortisol. Abbreviations: SAM, sympatho-adrenal-medullary; CRH, corticotropin-releasing hormone; AVP, arginine vasopressin; ACTH, adrenocorticotropic hormone.

Under stress, two major neuroendocrine systems orchestrate the physiological response: the sympatho-adrenal-medullary (SAM) axis and the hypothalamic-pituitary-adrenal (HPA) axis (Figure 1). The SAM initiates the immediate “fight or flight” response by secreting norepinephrine from postganglionic sympathetic nerves and secreting norepinephrine and epinephrine from the adrenal medulla. These catecholamines bind to α- and β-adrenergic receptors throughout the body, inducing rapid physiological changes such as increased heart rate, blood pressure, glycolysis, glycogenolysis, lipolysis, and oxygen consumption (Chu et al., 2024; Lyons et al., 2023; Steptoe and Kivimäki, 2012).

The HPA axis is responsible for a slower, sustained response. Stress signals activate the paraventricular nucleus of the hypothalamus, stimulating hypophysiotropic neurons to release corticotropin-releasing hormone (CRH) and arginine vasopressin (AVP) into the hypophysial portal system. AVP enhances the stimulatory effect of CRH on the anterior pituitary to secrete adrenocorticotropic hormone (ACTH), which in turn triggers the adrenal cortex to produce cortisol. Cortisol activates various physiological responses to help the body cope with stress. It also simultaneously exerts negative feedback on the HPA axis by binding to glucocorticoid receptors (GRs), thereby inhibiting the further secretion of CRH and ACTH (Abate et al., 2020; Dai et al., 2020; Lyons et al., 2023; Peckins and Beltz, 2020; Polsky et al., 2022). HPA and SAM are also involved in the regulation of several other biological processes, including immune function (Auger et al., 2024; Franco et al., 2019), energy production (Du et al., 2009), bone development (Zhou et al., 2013), and metabolism (Khani and Tayek, 2001). These pathways are highly regulated and essential for survival under acute conditions; however, chronic or repeated activation can dysregulate them, leading to detrimental effects on health.

## 3 Impact of chronic psychological stress on the hallmarks of aging

### 3.1 Mitochondrial dysfunction

Life stressors, particularly those experienced during early developmental stages, significantly impact mitochondrial function. Early adverse experiences, such as maltreatment or neglect, have been linked to persistent oxidative stress and low-grade inflammation (Duchowny et al., 2024; Gumpp et al., 2020). Mitochondria are key organelles within cells, responsible for producing energy through oxidative phosphorylation (OXPHOS) at the inner mitochondrial membrane. They contain electron transport chain (ETC) complexes I-IV and ATP synthase (complex V), which convert nutrients into adenosine triphosphate (ATP), the primary energy source for cellular activities. In this process, mitochondria also produce reactive oxygen species (ROS), which can function as signaling molecules but can also cause cellular damage if not adequately regulated. Therefore, maintaining redox balance is crucial for mitochondrial health and efficient energy production (Geldon et al., 2021).

Mitochondria play a key role in physiological responses to chronic psychological stress by influencing neuroendocrine, metabolic, and inflammatory pathways. Experimental deletion of mitochondrial genes alters HPA axis and SAM axis activity, catecholamine levels, and exaggerates the inflammatory response (Picard et al., 2015). Moreover, reduced expression of SIRT3 and PGC-1α, key regulators of mitochondrial bioenergetics and biogenesis, can further compromise mitochondrial activity and cellular energy metabolism (Scarpulla, 2011). Studies show that individuals with a history of early-life stress exhibit reduced ATP production and impaired mitochondrial respiratory capacity in immune cells and skeletal muscle (Duchowny et al., 2024; Gumpp et al., 2020). These mitochondrial impairments are observed not only in affected individuals but also in subsequent generations. Mitochondrial function may mediate the link between prenatal and postnatal stress exposures and neurodevelopmental outcomes in infants, with maternal mitochondrial bioenergetic profiles modulating early brain development and behavior (Zhao et al., 2022).

**FIGURE 2:**
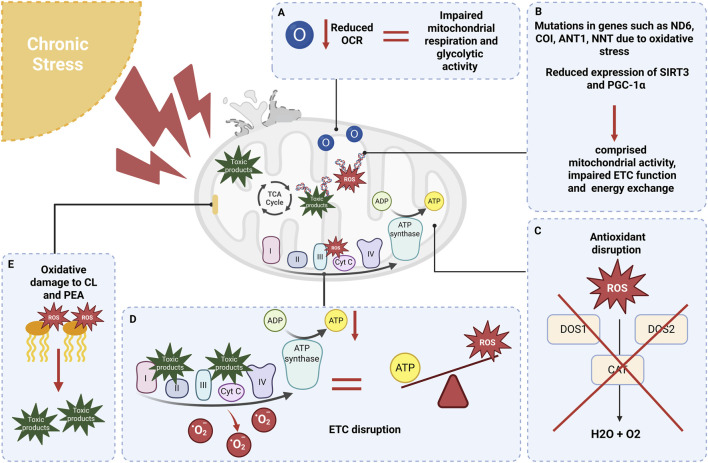
Summary of the effects of chronic stress on mitochondria. **(A)** Chronic stress reduces oxygen consumption rates, resulting in defective mitochondrial respiration and impaired glycolytic activity. **(B)** Stress increases the production of ROS and other harmful products, which consequently leads to an increase in mtDNA mutations. **(C)** Stress disrupts key antioxidant systems, such as CAT and SOD1 and SOD2, thereby increasing the prevalence of ROS. **(D)** Chronic stress impacts the ETC, leading to increased ROS production and decreased ATP synthesis. **(E)** The increased ROS generated by stress interacts with CL and PEA, resulting in disrupted membrane integrity. This disruption produces harmful byproducts that interfere with ETC proteins and contribute to mtDNA mutations. Abbreviations: ROS, reactive oxygen species; mtDNA, mitochondrial DNA; CAT, catalase; SOD1, superoxide dismutases 1; SOD2, superoxide dismutases 2; ETC, electron transport chain; ATP, adenosine triphosphate; CL, cardiolipin; PEA, phosphatidylethanolamine.

A primary mechanism by which chronic psychological stress impairs mitochondrial function is sustained ROS overproduction (Figure 2). Chronic stress reduces mitochondrial oxygen consumption rate (OCR) and disrupts ETC function, particularly at complex I, thereby promoting electron leakage and superoxide formation (Hunter et al., 2016; Nikolic et al., 2023; Yegorov et al., 2020). It also suppresses cellular antioxidant defenses. Clinical and preclinical studies have reported reduced activity of antioxidant enzymes, including SOD1, SOD2, and catalase (CAT), in depression (Herken et al., 2007; Qingfeng et al., 2019; Yıldız Miniksar and Göçmen, 2022). Consistent with this, nuclear factor erythroid 2-related factor 2 (Nrf2) regulates antioxidant-response gene expression and is downregulated under chronic stress (Zuo et al., 2022). Glucocorticoids can suppress the Nrf2 pathway *in vivo* and *in vitro* (Chen et al., 2020; Kratschmar et al., 2012), and *postmortem* analyses show lower Nrf2 expression in the frontal cortex of individuals with major depressive disorder (Martín-Hernández et al., 2018). Conversely, upregulation of Nrf2 alleviates depression-like behaviors, underscoring its protective role against stress-induced oxidative damage (Bansal et al., 2022; Yao et al., 2021).

Mitochondrial DNA (mtDNA) is a particularly vulnerable target of stress-induced oxidative damage because of its proximity to the ETC, absence of protective histones, and limited repair capacity (King and Copeland, 2025; Xu et al., 2025). Accumulating oxidative lesions in mtDNA can create a feed-forward cycle in which dysfunctional mitochondria generate more ROS, thereby exacerbating mtDNA damage and mitochondrial impairment. Consistent with this, oxidative stress induction in human epithelial cells significantly increased mitochondrial 8-oxo-2′-deoxyguanosine (8-oxodG), a widely used marker of oxidative DNA damage (Scala et al., 2023). *In vivo*, chronic mild stress impaired mitochondrial ultrastructure and function and reduced mtDNA integrity (Qingfeng et al., 2019). Moreover, oxidative damage to cardiolipin (CL) and phosphatidylethanolamine in the inner mitochondrial membrane can trigger lipid peroxidation via the isoprostane pathway, generating toxic byproducts that further compromise mtDNA integrity (Panov and Dikalov, 2020).

In addition to oxidative damage, chronic psychological stress disrupts mitochondrial quality control mechanisms. Under normal conditions, mitochondria undergo coordinated cycles of fusion and fission to maintain functional integrity and selectively eliminate damaged mitochondria via mitophagy or apoptosis. The PINK1/Parkin pathway, a well-characterized mitophagy mechanism, is impaired under chronic stress. Reduced PINK1 and Parkin expression has been observed in the hippocampus of a depressive mouse model, leading to diminished mitophagic activity (Yue et al., 2025). Activation of the PINK1/Parkin pathway in rats exposed to chronic restraint stress enhances mitophagy and restores mitochondrial quality control (Li et al., 2025). Impaired mitophagy can therefore promote the persistence of dysfunctional mitochondria and reinforce oxidative and cellular damage.

Importantly, the consequences of mitochondrial dysfunction directly intersect with multiple hallmarks of aging. Sustained mitochondrial ROS overproduction acts as a central mediator linking stress-induced mitochondrial damage to inflammation, genomic instability, telomere attrition, and cellular senescence. Excessive ROS not only damage mitochondrial components but also induce oxidative lesions in nuclear DNA (Mishra et al., 2009). Furthermore, mitochondrial dysfunction contributes to inflammation by facilitating the release of mtDNA and other danger-associated molecular patterns (DAMPs) into the cytosol and extracellular space, activating innate immune pathways such as cGAS-STING and the NLRP3 inflammasome (Kim J. et al., 2023). Elevated oxidative stress further accelerates telomere shortening by inducing oxidative base lesions and replication stress at chromosome ends, ultimately promoting cellular senescence (Metcalfe and Olsson, 2022).

Stress-induced impairment in mitochondrial function plays a critical role in the pathogenesis of age-related diseases. Psychological stress alters mitochondrial respiration and glycolysis, particularly in brain regions such as the prefrontal cortex (PFC) and substantia nigra (SN), which are implicated in the development of neurodegenerative diseases. In Parkinson’s disease (PD), oxidative stress can promote α-synuclein aggregation, which disrupts mitochondrial dynamics and contributes to neuronal degeneration (Banerjee et al., 2009). Genetic evidence further supports this link, as mutations in the mitophagy regulators PINK1 and Parkin are associated with familial early-onset PD (Lesage et al., 2020). *Postmortem* analyses further report mitochondrial bioenergetic defects in PD brain tissue, including impaired OXPHOS and increased mtDNA deletions. Notably, experimental delivery of damaged mtDNA into the brains of healthy mice induces PD-like neuropathology and functional deficits (Tresse et al., 2023). In Alzheimer’s disease, oxidative modifications to mitochondrial proteins impair ETC function, reducing ATP synthesis and increasing ROS production (Kriebel et al., 2020; Navarro and Boveris, 2004). Mitochondria-derived ROS increase amyloid-β levels, whereas lowering ROS levels decreases amyloid-β levels (Leuner et al., 2012). Beyond direct mitochondrial damage, stress-responsive nuclear receptors such as NR4A1 may further contribute to neurodegenerative vulnerability by reshaping mitochondrial function and neuronal energy homeostasis. NR4A1 regulates mitochondrial dynamics by modulating uncoupling proteins (Ucp2, Ucp4, and Ucp5). Sustained NR4A1 activation depletes ATP, activates AMP-activated protein kinase (AMPK), and promotes synaptic loss and neuronal atrophy (Jeanneteau et al., 2018; Sampath et al., 2017). The widespread impacts of stress-induced mitochondrial dysfunction are not limited to neurodegenerative diseases but also contribute to other age-related diseases (Somasundaram et al., 2024; Yang, 2025). In line with this central role, mitochondrial transplantation has shown therapeutic potential in preclinical models of PD, AD, and cardiovascular diseases by improving mitochondrial bioenergetics, reducing oxidative stress, and attenuating disease-related phenotypes (Eo et al., 2024; Gupta et al., 2025; Mokhtari et al., 2023).

### 3.2 Telomere attrition

Telomeres are repetitive short DNA sequences (TTAGGG), typically ranging from 5 to 15 kb, located at the ends of chromosomes where they function as protective caps essential for maintaining chromosomal integrity (Levstek et al., 2020). With each cell division, telomeres shorten due to the end-replication problem (Lulkiewicz et al., 2020). Before telomeres are entirely lost, they reach a critical length, known as the Hayflick limit, at which point cells can no longer divide. As a result, the cells either undergo apoptosis or exit the cell cycle and become senescent (Lyons et al., 2023). In certain cell types, such as stem cells and germ cells, telomere length is preserved by the enzyme telomerase, which adds repetitive DNA sequences to the ends of chromosomes. However, in most somatic cells, telomerase activity is insufficient to maintain telomere length, leading to gradual telomere erosion. As we age, telomeres progressively shorten, a phenomenon observed across multiple species (Whittemore et al., 2019). In humans, telomeres are estimated to shorten at a rate of approximately 20–40 base pairs per year (Brouilette et al., 2003; Daniali et al., 2013; Ye et al., 2023). The rate of telomere attrition is influenced by genetic, environmental, and lifestyle factors (Hecker et al., 2021).

Chronic psychological stress can exacerbate telomere shortening by increasing ROS production, primarily through mitochondrial dysfunction and inflammation. The overproduction of ROS leads to oxidative stress, which preferentially damages the guanine-rich telomeric regions, resulting in oxidative DNA lesions (Reichert and Stier, 2017). In addition, oxidative stress can cause single-strand breaks in telomeres (von Zglinicki et al., 2000) and halt the replication fork, leading to telomere erosion (Coluzzi et al., 2019; Jurk et al., 2014).

The connection between psychological stress and telomere length has attracted substantial attention following a study by Epel et al. (2004), which demonstrated that healthy women caring for chronically ill children had significantly shorter telomeres and lower telomerase activity than mothers of healthy children, independent of age. Moreover, the study showed that telomere length in caregiving mothers was negatively correlated with the perceived duration of stress; the longer the caregiving stress, the shorter the telomeres and the lower the telomerase activity. Subsequent research confirmed these findings. For example, individuals providing care for a spouse or parent with dementia had significantly shorter telomere length compared to those without caregiving responsibilities (Kiecolt-Glaser et al., 2010). Other studies have linked various life stressors and adversities, such as childhood physical neglect (Vincent et al., 2017), institutional discrimination (Thomas et al., 2021), and Socioeconomic deprivation (Powell-Wiley et al., 2020), with shortened telomere length. Chronic psychological stress can lead to dysregulation of the stress response and elevated cortisol levels (Karin et al., 2020). Elevated hair cortisol concentration in individuals with previous child welfare residential placements and a history of childhood maltreatment is associated with shorter leukocyte telomere length (Bürgin et al., 2022).

*In vitro*, studies have demonstrated that exposure of peripheral blood mononuclear cells (PBMCs) to cortisol significantly shortens telomere length (Vartak et al., 2014). Additionally, cortisol exposure decreases telomerase activity in PBMCs (Choi et al., 2008). Interestingly, prolonged exposure to cortisol does not affect telomere length in human fibroblasts (Zannas et al., 2020).

Genetics also plays a significant role in regulating telomere length. Several studies have confirmed that telomere length is a highly heritable trait (Andreu-Sánchez et al., 2022; Guo et al., 2022; Lee et al., 2014), and a strong association exists between parental and offspring telomere length (Broer et al., 2013; Chen et al., 2022; Njajou et al., 2007). Some studies have suggested a possible association between maternal psychological stress during pregnancy and shortened telomere length in newborns (Bosquet Enlow et al., 2021; Entringer et al., 2013; Khalil et al., 2023; Marchetto et al., 2016; Salihu et al., 2016). However, a major limitation of these studies is the relatively small sample sizes. Contrarily, larger studies have reported no significant association between maternal stress and newborn telomere length (Ämmälä et al., 2020; Izano et al., 2020; Naudé et al., 2023). The relationship between parental psychological stress and offspring telomere length remains inconclusive, with inconsistent findings across studies.

### 3.3 Cellular senescence

Cellular senescence is the irreversible arrest of cellular proliferation, typically triggered by various biomolecular changes, including DNA damage, metabolic stress, mitochondrial dysfunction, oxidative stress, and telomere shortening (Campisi and d’Adda di Fagagna, 2007; Frasca et al., 2021). Cellular senescence is considered a protective mechanism that halts the propagation of damaged cells that could otherwise develop into cancer (Mijit et al., 2020). Two tumor suppressor pathways mainly regulate this process: the p53/p21 pathway and the p16^INK4A^/pRB pathway. Activation of one or both of these pathways results in cell cycle arrest (Roger et al., 2021).

Senescent cells are characterized by enlarged, flattened morphology, chromatin remodeling, metabolic alterations, resistance to apoptosis, and the secretion of a senescence-associated secretory phenotype (SASP) (Huang et al., 2022; Kumari and Jat, 2021). The load of senescent cells is higher in older individuals than in younger ones, although senescence rates vary across cell types (Tuttle et al., 2020). As senescent cells accumulate within tissues, they impair normal cellular function and contribute to the development of age-related diseases (Calcinotto et al., 2019).

Chronic psychological stress induces cellular senescence through several potential mechanisms, primarily by promoting oxidative stress and telomere shortening. These stress-induced cellular changes can activate the DNA damage response (DDR), a key pathway leading to cellular senescence (Feringa et al., 2018). DDR activates kinases such as ATM (ataxia-telangiectasia mutated) and ATR (ataxia-telangiectasia and Rad3-related), which phosphorylate p53, stabilizing it and promoting its transcriptional activity. Activated p53 induces p21 expression, leading to cell cycle arrest. Another critical pathway involved in stress-induced senescence is the p16^INK4A^/pRB pathway. p16^INK4A^ binds to CDK4/6, preventing the phosphorylation of the retinoblastoma protein (Rb), thereby suppressing E2F transcription factors and enforcing cell cycle arrest (Mansfield et al., 2024).

Several studies have linked psychological stress with the upregulation of key senescence markers. In an *in vivo* model, mice exposed to a chronic unpredictable stress paradigm for 2 months to induce depression-like symptoms exhibited increased protein expression of p16^INK4A^ and SA-β-gal in the hippocampus of stressed mice (Lin et al., 2021). Another study showed that mice subjected to chronic social stress exhibited elevated levels of p16^INK4A^ and p21 in various organs, as well as signs of DNA damage in hippocampal and cortical neurons (Lyons et al., 2025).

In humans, p16^INK4a^ expression is significantly upregulated in individuals with major depressive disorder (Teyssier et al., 2012). In stressful conditions such as sleep deprivation, p16^INK4a^ expression levels are elevated in individuals who experienced partial sleep deprivation (Carroll et al., 2016). Additionally, individuals with higher exposure to chronic stressors such as relationship conflicts, work-related stress, and financial strain showed increased expression levels of p16^INK4a^ (Rentscher et al., 2019). In a clinical study, tendon biopsies from patients treated with glucocorticoid injections for rotator cuff tendinopathy exhibited a significant increase in the percentage of p53 and p21 positive cells in biopsies obtain 7 weeks after treatment, compared to pre-injection samples from the same individuals, suggesting *in vivo* activation of the p53/p21 pathway (Poulsen et al., 2014).

Chronic Inflammation is another mechanism mediating psychological stress-induced senescence. The nuclear factor kappa B (NF-κB) is a central regulator of SASP and can be triggered by oxidative stress. The activation of the NF-κB pathway induces the production of pro-inflammatory SASP factors, which not only reinforce the senescent state but also promote paracrine senescence in neighboring healthy cells (Nelson et al., 2018).

A study showed that dexamethasone (a synthetic glucocorticoid) treatment induces cellular senescence in bone marrow adipocyte (BMAd) lineage cells isolated from adult mice. Glucocorticoid-induced senescence spread to neighboring bone and bone marrow cells via SASP factors. Furthermore, the treatment increases the synthesis of SASP factors, including oxylipins, particularly prostaglandin D2 (PGD2), which activates peroxisome proliferator-activated receptor gamma (PPARγ). PPARγ, in turn, upregulates the expression of key senescence genes, including p16^INK4a^ and p27^KIP1^ (Liu et al., 2023). A similar PPARγ-driven induction of senescence has been reported in human cells (Gan et al., 2008).

### 3.4 Epigenetic alterations

Epigenetics refers to chemical modifications to DNA or histones that influence gene expression without altering the underlying DNA sequence, including DNA methylation and histone modifications. These alterations can be influenced by environmental and lifestyle factors, such as stress, physical activity (Fiorito et al., 2021), diet (Kim et al., 2022), smoking (Klopack et al., 2022a), and alcohol consumption (Nannini et al., 2023). The current understanding of the interplay between chronic psychological stress, epigenetic regulation, and aging suggests that chronic psychological stress induces epigenetic alterations, which, in turn, dysregulate gene expression profiles crucial for maintaining cellular and systemic homeostasis.

Aging is associated with distinct alterations in epigenetic signatures, particularly changes in DNA methylation. As a result, age-related DNA methylation patterns have been proposed as biomarkers of biological aging. In 2013, Steve Horvath developed the first “epigenetic clock” by identifying a set of CpG sites whose methylation levels strongly correlate with chronological age (Horvath, 2013). Since then, multiple epigenetic clocks have been developed to predict biological age and health outcomes, providing an opportunity to evaluate the impact of psychological stress on epigenetic aging.

Several studies have investigated the association between psychological stress and epigenetic age acceleration. Fiorito et al. (2017) demonstrated that low socioeconomic status (SES) is associated with accelerated epigenetic aging across three cohort studies involving over 5,000 individuals. Participants with low SES showed an estimated 1-year acceleration in biological age compared to those with high SES. A recent cohort study of 3,963 participants from the National Longitudinal Study of Adolescent to Adult Health, a U.S.-based longitudinal population study, found an association between familial loss (loss of a parent, sibling, child, or partner/spouse) and epigenetic alterations associated with aging Moreover, individuals who experienced two or more familial losses showed greater biological age acceleration than those with one or no losses. The study found that familial losses occurring during adulthood were associated with accelerated biological aging, whereas losses during childhood or adolescence did not appear to influence biological age in adulthood. These findings suggest that the impact of familial loss on epigenetic aging may be reversible or age-dependent (Aiello et al., 2024).

Other types of psychological stressors, including violence exposure (Jovanovic et al., 2017), racial discrimination (Cuevas et al., 2024; Ruiz-Narváez et al., 2024), and traumatic experience (Boks et al., 2015), have also been linked to accelerated biological aging. However, some studies have reported no association between psychological stress and accelerated biological aging. For example, Vetter et al. found no significant association between perceived psychological stress and epigenetic age acceleration in older individuals (mean age, 75.6 years) across five epigenetic clocks (Vetter et al., 2022). Similarly, a study involving 1,762 women from the ALSPAC and NSHD cohorts found no association between childhood adversities, including socioeconomic status, and various types of cumulative psychosocial adversity, with epigenetic age acceleration in adulthood, except in cases involving sexual abuse (Lawn et al., 2018).

Although growing evidence supports a link between psychological stress and epigenetic age acceleration, findings across studies remain inconsistent. Several factors may explain these discrepancies. First, epigenetic clocks are based on different sets of CpG sites, and while some clocks may capture stress-related epigenetic changes, others may not. This variability has been observed across multiple studies in which associations were identified with certain clocks but not others (Hamlat et al., 2021; Joshi et al., 2023; Klopack et al., 2022b; Marini et al., 2020; Rampersaud et al., 2022). Second, individual differences such as psychological resilience and self-control may mediate the effects of psychological stress on epigenetic aging. It has been shown that individuals with higher levels of self-control and resilience exhibit slower stress-induced epigenetic aging compared to those with lower levels (Fogelman et al., 2021; Zhang et al., 2023). Third, the timing of stress exposure appears to influence its impact on epigenetic aging. Recent studies suggest the existence of sensitive periods during which psychological stress may have a more pronounced effect on epigenetic aging (Chang et al., 2024; Lussier et al., 2023).

Beyond DNA methylation changes captured by epigenetic clocks, stress exposure induces methylation changes in genes that regulate the stress response, including NR3C1, which encodes the glucocorticoid receptor (GR), and FK506 binding protein 51 (FKBP5), a co-chaperone of GR that influences receptor sensitivity. Hypermethylation of NR3C1 reduces GR expression and decreases cortisol sensitivity (Kolb et al., 2023). Several studies have demonstrated that exposure to chronic psychological stress is associated with increased methylation on NR3C1. For example, children with a history of maltreatment have been found to exhibit increased methylation of the NR3C1 promoter region compared to non-maltreated children (Cicchetti and Handley, 2017). Similarly, in a *postmortem* study, hippocampal tissue from suicide victims with a history of childhood abuse showed increased methylation at the NR3C1 promoter, along with decreased expression of GR mRNA (McGowan et al., 2009).

Elevated FKBP5 levels reduce GR sensitivity to cortisol, disrupting negative feedback mechanisms and leading to sustained activation of the HPA axis and elevated circulating cortisol levels (Häusl et al., 2021; Zannas et al., 2016). FKBP5 expression increases with age in both mice (Sabbagh et al., 2014) and humans (Matosin et al., 2023) and has been linked to psychiatric disorders (Binder et al., 2008; Matosin et al., 2023). Early life stress and childhood trauma have been found to accelerate the age-associated decrease in FKBP5 methylation in peripheral blood (Zannas et al., 2019). Decreased methylation at two CpG sites in intron 7 of FKBP5 has also been reported in maltreated children (Tyrka et al., 2015). A *postmortem* study found increased FKBP5 mRNA and protein expression and lower methylation at the FKBP5 promoter in the prefrontal cortex and hippocampus of suicide victims, most of whom had psychiatric disorders (Rizavi et al., 2023). Additionally, Everson et al. (2023) reported decreased FKBP5 promoter methylation in buccal and blood cells of children with abusive injuries compared to children with accidental injuries. These epigenetic dysregulations of NR3C1 and FKBP5 lead to the dysregulation of the HPA axis (Sharan and Vellapandian, 2024).

### 3.5 Inflammation

One biological mechanism linking chronic psychological stress to accelerated aging is the inflammatory pathway (Franceschi and Campisi, 2014; Liu et al., 2017). Accumulating evidence indicates that psychological stress can activate inflammatory responses in the brain and peripherally through interactions between the neuroendocrine and immune systems (Calcia et al., 2016; Rohleder, 2014). Physiologically, our body responds to stress stimuli in a state called “allostasis,” in which stress response pathways are activated, leading to the release of chemical mediators such as glucocorticoids (GCs), adrenaline, and noradrenaline, which evoke a defensive response. In addition, GCs are known to suppress immune cell activity, reduce the production of pro-inflammatory cytokines, such as tumor necrosis factor alpha (TNF-α) and interleukin-6 (IL-6), and promote anti-inflammatory cytokines, such as interleukin-10 (IL-10) and Transforming Growth Factor beta (TGF-β) (Maydych, 2019; Miller et al., 2002). This is a protective mechanism that prevents an overactive immune response during a stressful event, allowing the body to focus its resources on immediate survival.

However, when stress stimuli are prolonged, it can lead to chronic “allostasis,” which contributes to pathophysiology and stress-related diseases. Chronic elevation of cortisol and related hormones leads immune cells to become less sensitive to the anti-inflammatory effects of glucocorticoids, thereby impairing their ability to suppress inflammation (Pariante, 2017). This state, known as glucocorticoid resistance, enables the continued production of pro-inflammatory cytokines, such as IL-6, TNF-α, and interleukin-1 beta (IL-1β), despite high cortisol levels (Miller et al., 2002). Pérez-Nievas et al. (2007) found that, after acute immobilisation stress, rats with higher baseline plasma corticosterone levels accumulated more prostaglandin E2 (PGE2) and showed a weaker anti-inflammatory response. Further study revealed that parents of children with cancer, experiencing chronic stress, showed decreased immune cell sensitivity to dexamethasone in inhibiting IL-6 production (Miller et al., 2002). Furthermore, Pace et al. (2006) found that male patients with major depression and increased early life adversity (ELA) exhibited significantly increased activation of IL-6/NF-κB pathway in response to the Trier Social Stress Test compared to those who were not, with these inflammatory markers correlating with depression severity. These results were in line with several studies that have associated exposure to ELA with increased inflammation in later life (Coelho et al., 2014; Danese et al., 2008; Kiecolt-Glaser et al., 2010; Maydych, 2019).

Within the brain, psychological stress triggers a cascade of neuroimmune interactions, especially involving microglia, the brain’s resident immune cells. Chronic stress can also dysregulate the sympathetic nervous system activity, with norepinephrine acting via adrenergic receptors on microglia to stimulate the production and release of inflammatory mediators, such as IL-6 and TNF-α, through MAPK pathway activation and enhancement of inflammasome components (Carrier et al., 2021; Huang et al., 2012; Liu et al., 2017). Simultaneously, elevated norepinephrine mobilizes monocytes from the bone marrow into the bloodstream. Sustained microglial activation recruits peripheral monocytes. Increased brain macrophages and circulating monocytes contribute to further elevated levels of pro-inflammatory cytokine production in the brain (Wohleb and Delpech, 2017). This further amplifies neuroinflammatory signaling, disrupts microglia–neuron communication, impairs microglial phagocytosis, and contributes to oxidative stress and synaptic dysfunction (Şahin and Aslan, 2024). Collectively, these findings suggest that prolonged stress impairs the body’s natural anti-inflammatory processes, potentially increasing vulnerability to inflammation-related diseases.

Importantly, while psychological stress can trigger inflammation, elevated inflammatory activity can, in turn, promote stress-related symptoms and psychological disorders. Several studies have been positively associated with proinflammatory cytokine levels and stress-related symptoms such as depression, anxiety, and cognitive impairment (Liu et al., 2017; Maydych, 2019). This bidirectional relationship reflects the complex communication between the immune system and the central nervous system. Proinflammatory cytokines, including IL-1β, IL-6, and TNF-α, can signal to the brain through humoral, neural, and cellular pathways, ultimately affecting neurotransmission, neuroendocrine activity, and neural circuits implicated in mood and cognition (Yang et al., 2022). Neuroimaging studies have shown that elevated levels of these cytokines are associated with increased activation of the amygdala and prefrontal cortex, brain regions involved in threat processing, emotional regulation, and executive function (Inagaki et al., 2012; Muscatell et al., 2015). As a result, individuals exposed to chronic inflammation are more sensitive to negative emotional stimuli and have an increased risk for symptoms such as persistent anxiety, anhedonia, and cognitive slowing (Rhie et al., 2020).

The clinical phenomenon of “sickness behavior” is a striking example: acute or chronic inflammation from infection, autoimmune disease, or experimental immune activation can induce fatigue, social withdrawal, reduced motivation, and low mood symptoms closely resembling those in depressive and anxiety disorders (Henry et al., 2008; Liu et al., 2017). Experimental studies have shown that administering proinflammatory cytokines, vaccines, or bacterial endotoxin to healthy participants rapidly induces a negative mood, heightens attentional bias toward threatening or negative stimuli, and impairs cognitive performance (Benson et al., 2017). Importantly, individuals with existing mood disorders often display exaggerated inflammatory responses to psychological stress, suggesting a vulnerability to both heightened inflammation and psychological distress (Mucci et al., 2020).

Inflammation can also potentiate stress sensitivity by altering the regulation of the HPA and SAM axes, which are core mediators of the body’s response to psychological challenges (Liu et al., 2017). Chronic inflammatory signaling disrupts feedback mechanisms within these systems, leading to heightened baseline anxiety, increased perceived stress, and exaggerated stress responses to even minor environmental challenges (Dooley et al., 2018). This creates a self-perpetuating loop in which inflammation and psychological stress continually reinforce each other, increasing vulnerability to mood and anxiety disorders and compounding the risk of chronic disease (Nelles et al., 2025).

Cognitive functions are directly involved in this process. Inflammatory activity is associated with negative attentional bias, greater focus on and difficulty disengaging from negative or threatening information, which is a hallmark of depression and anxiety. Such biases reinforce negative mood, promote rumination, and reduce the capacity to recover from stress (Benson et al., 2017; Boyle et al., 2017).

Societal and environmental factors can further amplify these interactions. Chronic discrimination, socioeconomic hardship, and poor social support are each associated with elevated proinflammatory markers and greater risk of psychological stress and mood disorders. When systemic inflammation is heightened by social adversity, the psychological toll of stress is magnified, illustrating how biological and psychosocial pathways converge to influence mental health outcomes (Androulakis, 2024).

### 3.6 Genomic instability

Chronic psychological stress accelerates biological aging by inducing DNA damage. This process is mediated by multiple interconnected mechanisms, including oxidative stress, inflammation, dysregulation of the HPA axis, epigenetic alterations, and impaired DNA repair systems (Gaffey et al., 2016). These mechanisms collectively disrupt cellular homeostasis, compromise genomic stability, and increase susceptibility to age-related diseases (Jurk et al., 2014).

One of the principal mechanisms through which chronic psychological stress induces DNA damage is oxidative stress. Under persistent stress conditions, there is an overproduction of ROS, which are chemically reactive molecules capable of damaging cellular structures, including nucleic acids. The interaction between ROS and DNA results in base modifications, single- and double-strand breaks, and the formation of oxidized nucleosides such as 8-oxo-dG (Goh et al., 2021). This oxidative stress is further intensified by the upregulation of ROS-producing enzymes, such as NADPH oxidase 4 (NOX4), and the downregulation of antioxidant defense mechanisms involving SOD1 and SOD2, thereby reducing the cell’s capacity to neutralize ROS (Boonpraman and Yi, 2023; Dokic et al., 2012).

In addition to oxidative stress, chronic psychological stress activates inflammatory pathways that further contribute to DNA damage. The NF-κB signaling pathway is commonly activated by stress and plays a crucial role in regulating the expression of pro-inflammatory genes. Activation of this pathway leads to the release of cytokines such as IL-6 and TNF-α, both of which promote oxidative stress and interfere with DNA repair mechanisms (Lam et al., 2022; Picard et al., 2015). TNF-α, for instance, has been shown to suppress the function of 8-oxoguanine DNA glycosylase (OGG1), an essential enzyme in the base excision repair (BER) pathway that repairs oxidative DNA lesions (Hassa and Hottiger, 2008). Furthermore, inducible nitric oxide synthase (NOS2) can produce reactive nitrogen species (RNS), which exacerbate DNA damage through nitration and deamination reactions, leading to mutagenic lesions (Flaherty et al., 2017).

Another critical pathway affected by chronic psychological stress is the HPA axis. Prolonged activation of this axis results in sustained cortisol secretion. Cortisol modulates the expression of several DNA repair genes and has been implicated in the suppression of genes such as BRCA1 and RAD51, which are vital for homologous recombination (HR) repair of double-strand breaks (Werner, 2022). Cortisol also modulates FKBP5 expression, further influencing the genomic stress response (Fries et al., 2017). In addition, cortisol reduces the expression of XRCC1 and APE1, genes critical for the base excision repair and DNA damage response pathways, thereby diminishing cellular ability to address DNA insults) Clementi et al., 2020).

Empirical evidence from both animal and human studies supports these molecular findings. In an *in vivo* study in mice, Jurk et al. (2014) observed that chronic inflammation associated with psychological stress led to marked telomere dysfunction, accumulation of DNA damage, and downregulation of key DNA repair genes. These changes were related to features of accelerated aging. Human studies have provided comparable results. A longitudinal study involving caregivers of chronically ill patients found elevated levels of IL-6 and markers of oxidative DNA damage, coupled with reduced DNA repair capacity, in caregivers compared with non-caregiver controls (Kiecolt-Glaser et al., 2003).

Moreover, chronic psychological stress induces epigenetic alterations that further compromise DNA integrity. Epigenetic modifications, particularly changes in histone acetylation and methylation, affect chromatin structure and gene expression. These changes can limit the accessibility of DNA repair enzymes to damaged sites and enhance the vulnerability of genomic regions to damage (Zannas et al., 2015). For example, stress-related epigenetic repression of p53, a gene central to cell cycle regulation and DNA repair, may impair the cellular capacity to respond to genotoxic stress, increasing the risk of mutagenesis and disease progression (Busbee et al., 2022).

**FIGURE 3:**
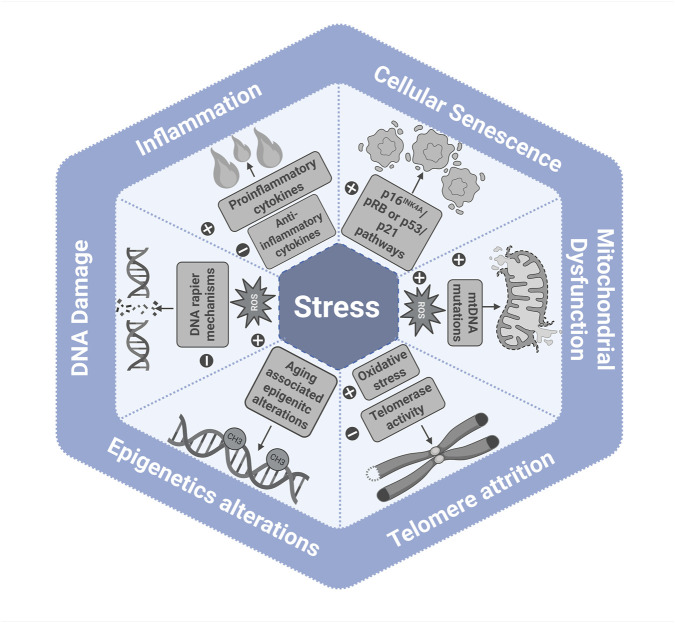
Summary of mechanistic links between psychological stress and aging.

Altogether, these are the current evidence of the relationship between chronic psychological stress and aging. Chronic stress utilizes mitochondrial dysfunction, oxidative stress, telomere attrition, cellular senescence, epigenetic alterations, inflammation, and genomic instability to accelerate aging and increase vulnerabilities to age-related diseases (Figure 3).

## 4 Interventions

The effectiveness of physiological stress interventions depends critically on early stress detection to support timely intervention before long-term biological wear occurs. Recent advances in wearable biosensor technology have enabled real-time assessment and continuous monitoring of key stress indicators, including cardiovascular measures (e.g., heart rate, blood pressure) and autonomic nervous system markers (e.g., electrodermal activity) (Pinge et al., 2024). Additionally, biochemical markers measured in biofluids (e.g., cortisol, inflammatory cytokines) provide insight into stress response pathway activity and stress-related immune activation (Budala et al., 2025). Several salivary biomarkers have been proposed for stress detection. These include cortisol, the primary effector hormone of the HPA axis (Durán-Carabali et al., 2021); salivary α-amylase, a marker for SAM axis activation (Santos et al., 2021; Schneider et al., 2025); chromogranin A, which reflects sympathetic nervous system activity (Deneva et al., 2026); and elevated IL-6 levels can indicate stress-related immune activation (Koreki et al., 2024). Beyond salivary markers, circulating cell-free mitochondrial DNA (cf-mtDNA) has been proposed as a biomarker of stress-induced mitochondrial damage, with studies reporting increases in cf-mtDNA following stress exposure in humans (Chattopadhyay et al., 2025; Trumpff et al., 2019). Importantly, integrating biomarker monitoring with intervention strategies can provide significant insights into treatment response and effectiveness (Eilertsen et al., 2025; Fuhrmann et al., 2025). Various intervention strategies have been proposed to combat chronic physiological stress and promote healthy aging. These include lifestyle interventions, psychological and behavioral interventions, medical and therapeutic interventions, and multi-component and community-based interventions.

### 4.1 Lifestyle interventions

#### 4.1.1 Physical activity

The promotion of physical exercise has been proposed to reduce the risk of developing aging-associated diseases (Langhammer et al., 2018). Additionally, physical activity is associated with improvements in mental health, delays the onset of dementia, and enhances the quality of life and wellbeing (Schuch et al., 2016; Livingston et al., 2017; Langhammer et al., 2018). Research has shown that physical activity has a significant positive impact on healthy aging, reducing the likelihood of health decline in older adults. This conclusion was drawn from a large, harmonized dataset comprising eight aging cohorts from various countries, including Australia, the United States, Mexico, Japan, South Korea, and several European nations, involving 130,521 participants aged approximately 63 years on average. Over a follow-up period of up to 10 years, researchers identified three distinct trajectories of healthy aging: a high-stable group (71.4%), a low-stable group (25.2%), and a high-initial-health group with rapid decline (3.4%). The study showed that engaging in any level of physical activity was associated with significantly lower odds of belonging to the low-stable or fast-decline groups compared with the high-stable group. These associations remained consistent across different measures of physical activity and across various sensitivity analyses. Additionally, the study highlights the importance of promoting physical activity as a key strategy in public health policies to support healthy aging and prevent functional decline and disability (Moreno-Agostino et al., 2020). Moreover, both short- and long-term physical exercise can significantly increase TERT gene expression and telomerase activity, key mechanisms that maintain telomere length and genomic stability, which are crucial for healthy aging. This systematic review and meta-analysis, which included human and rodent studies, revealed that a single bout of exercise and long-term exercise training upregulate telomerase activity in non-cancerous somatic cells. Endurance athletes also showed higher leukocyte TERT expression and telomerase activity than inactive individuals. Despite heterogeneity across studies, the overall findings support regular physical activity as an effective and affordable strategy to attenuate telomere shortening, thereby potentially extending health span and promoting longevity through telomerase-dependent pathways (Denham and Sellami, 2021).

#### 4.1.2 Nutrition

Chronic psychological stress contributes to neuroinflammation, oxidative damage, metabolic imbalance, and cognitive decline, all of which accelerate the progression of age-related diseases (Kivimäki et al., 2023). Nutrition offers a protective mechanism through multiple pathways: Diets rich in polyphenols, such as those found in fruits, vegetables, legumes, nuts, and olive oil, act as powerful antioxidants and anti-inflammatory agents, neutralizing ROS and regulating immune responses, thereby reducing neuroinflammation linked to Alzheimer’s disease and other cognitive impairments (Obrenovich et al., 2011). The grape polyphenols, for instance, improve insulin sensitivity, cardiovascular health, and neuroprotection, with studies showing enhanced motor and memory functions in aged rats and reduced cognitive degradation in rodents fed polyphenol-rich berry extracts (Bensalem et al., 2019; Nassiri-Asl and Hosseinzadeh, 2009; Shukitt-Hale et al., 2006). Furthermore, dietary patterns like the Mediterranean, Dietary Approaches to Stop Hypertension (DASH), and Mediterranean-DASH Intervention for Neurodegenerative Delay (MIND) diets, which emphasize plant-based foods, whole grains, lean protein, healthy fats, and minimal intake of processed foods and saturated fats, have been strongly associated with reduced risk of chronic diseases such as cardiovascular disease, diabetes, and neurodegenerative disorders (Liu et al., 2025; Kheirouri and Alizadeh, 2022; Shang et al., 2023). These diets also enhance memory, attention, and executive functions while lowering depression and anxiety, which are exacerbated by chronic stress (Khayyatzadeh et al., 2018; Kheirouri and Alizadeh, 2022; Shang et al., 2023). The incorporation of polyunsaturated fatty acids, especially Eicosapentaenoic acid (EPA) and Docosahexaenoic acid (DHA) from fatty fish, is essential for brain development and cognitive performance across the lifespan (Øyen et al., 2018). Additionally, caloric restriction (CR), when implemented without compromising micronutrient intake, has been shown to improve glucose metabolism, reduce inflammation, lower oxidative stress, and promote metabolic homeostasis (Flanagan et al., 2020). The CALERIE trial, for example, demonstrated that a 25% reduction in caloric intake over 2 years led to improvements in cardiometabolic markers and weight loss in healthy individuals. Altogether, the research emphasizes that a nutrient-dense, anti-inflammatory dietary approach, through specific food choices, macronutrient balance, and controlled caloric intake, not only reduces the biological burden of chronic psychological stress but also strengthens physiological and cognitive resilience, contributing to healthier aging and improved quality of life (Rickman et al., 2011).

#### 4.1.3 Sleep hygiene

Sleep hygiene plays a pivotal role in reducing chronic psychological stress and promoting healthy aging by enhancing the quality, duration, and restorative function of sleep. Aging is often accompanied by changes in sleep architecture, including reduced Rapid Eye Movement (REM) sleep and slow-wave sleep, increased nighttime awakenings, and decreased total sleep time (Gokcen, 2023). These alterations are linked to cognitive decline, emotional instability, and frailty (Gokcen, 2023; Li et al., 2018). Sleep hygiene, defined as behavioral and environmental strategies that support better sleep, can mitigate these age-related changes and serve as a non-pharmacological tool to support mental and physical health in the elderly (Baranwal et al., 2023). Poor sleep quality has been associated with heightened psychological stress, increased cortisol levels, and impaired emotional regulation. Interventions focused on sleep hygiene, such as maintaining a consistent sleep schedule, reducing caffeine and alcohol intake, and optimizing the sleep environment, have demonstrated improvements in sleep quality, reduced daytime sleepiness, and enhanced cognitive performance (Almondes et al., 2017). For instance, older adults practicing sleep hygiene showed significant gains in attention, memory, and executive functioning, contributing to preserved autonomy and wellbeing. Moreover, improved sleep supports glymphatic clearance of neurotoxins and reduces inflammatory processes, both of which are crucial for protecting against neurodegenerative diseases and systemic illnesses (Baranwal et al., 2023). The bidirectional relationship between sleep and psychological wellbeing is well-documented; sleep deprivation exacerbates stress, depression, and anxiety, while adequate sleep fosters resilience and emotional balance (Béphage, 2005). Collectively, the literature supports the notion that promoting sleep hygiene in older adults not only alleviates chronic psychological stress but also contributes significantly to healthy aging by preserving cognitive function, emotional regulation, and physiological resilience. Therefore, integrating sleep hygiene into routine geriatric care and public health strategies may offer a simple yet powerful approach to enhance the quality of life and healthspan in aging populations. This underscores the need for further research and implementation of sleep-focused interventions in elderly care settings (Almondes et al., 2017; Baranwal et al., 2023; Béphage, 2005; Gokcen, 2023; Li et al., 2018).

#### 4.1.4 Social engagement

Social engagement plays a vital role in reducing chronic psychological stress and promoting healthy aging by enhancing emotional wellbeing, cognitive resilience, and life satisfaction. Research consistently shows that participation in social activities, such as volunteering, community programs, and social gatherings, fosters a sense of connection and purpose in older adults, which directly mitigates psychological distress (Monteiro et al., 2025). Mackenzie and Abdulrazaq demonstrated that social engagement mediates the relationship between participation in social activities and psychological distress among older adults. Specifically, their findings revealed that increased participation in social activities enhances social engagement, defined by the frequency and quality of interactions with close friends and family, which in turn reduces symptoms of anxiety and depression (Mackenzie and Abdulrazaq, 2021). This is supported by socioemotional selectivity theory, which suggests that older adults tend to prioritize emotionally meaningful relationships, leading to more satisfying and protective social interactions. Moreover, Kim et al. (2021) emphasized that psychological and social wellbeing are key targets in resilient aging, and interventions aimed at enhancing social connectedness contribute significantly to maintaining mental health in older populations. Social support, particularly perceived emotional availability from trusted others, serves as a psychological buffer during times of stress, reducing cortisol levels and promoting emotional regulation. Together, these findings underscore the importance of fostering social engagement through structured social participation, not merely as a lifestyle enhancement, but as a core strategy to reduce psychological stress and delay cognitive and functional decline in aging populations. Public health initiatives and geriatric care should therefore prioritize social connectivity as a preventive and therapeutic tool to promote holistic and healthy aging.

### 4.2 Psychological and behavioral interventions

#### 4.2.1 Cognitive behavioral therapy

Cognitive Behavioral Therapy (CBT) plays a vital role in mitigating chronic psychological stress and promoting healthy aging, particularly in older adults. Numerous studies underscore the efficacy of CBT in addressing depression, anxiety, and stress-related disorders in this population, which are commonly linked with deteriorating physical health, cognitive decline, and social withdrawal (LaRocca and Scogin, 2015; Palazzolo, 2015; Оверчук, 2025). CBT operates by helping individuals reframe negative thought patterns, develop adaptive coping mechanisms, and engage in goal-oriented behaviors that improve psychological resilience and quality of life (LaRocca and Scogin, 2015; Palazzolo, 2015). Research has shown that CBT significantly alleviates depressive and anxiety symptoms in elderly patients, contributing to improved social functioning, reduced healthcare usage, and enhanced feelings of wellbeing. Palazzolo reported that CBT is as effective as pharmacotherapy in treating late-life depression and anxiety, with the added benefit of fewer side effects and longer-lasting benefits post-treatment. Importantly, CBT can be adapted for the cognitive and sensory changes in aging adults by using simplified content, slower pacing, and including caregivers as co-therapists when appropriate (Palazzolo, 2015). Moreover, CBT has been shown to positively influence biological markers of aging. Another study shows that CBT reduces chronic stress by regulating cortisol levels and decreasing inflammatory markers, both of which are associated with accelerated aging. These effects help preserve telomere length, a key biomarker of cellular aging, thereby potentially delaying age-related diseases such as cardiovascular disease and neurodegeneration (Оверчук, 2025). Additionally, CBT has been shown to enhance psychosocial factors vital for healthy aging, including self-esteem, interpersonal relationships, and a sense of purpose. LaRocca and Scogin emphasized that when combined with strong social support, CBT further improves quality of life in rural older adults, suggesting a synergistic effect between psychological therapy and environmental factors (LaRocca and Scogin, 2015).

#### 4.2.2 Mindfulness and meditation

Mindfulness and meditation have emerged as powerful non-pharmacological tools in promoting healthy aging and mitigating chronic psychological stress. Mindfulness practices, encompassing focused attention, open monitoring, and loving-kindness meditation, are shown to enhance cognitive, emotional, and physiological wellbeing among older adults (Fountain-Zaragoza and Prakash, 2017; Lutz et al., 2021; Tang et al., 2015). These practices cultivate present-moment awareness and non-reactivity, which help reduce stress and enhance emotion regulation, particularly valuable for aging populations facing cognitive decline and social isolation (Fountain-Zaragoza and Prakash, 2017). Empirical evidence suggests that mindfulness meditation significantly improves attentional control, mitigates age-related deficits in executive functioning, and supports the maintenance of goal-directed attention. These cognitive benefits are accompanied by reductions in systemic inflammation and psychological distress, which contribute to improved physical health and quality of life (Fountain-Zaragoza and Prakash, 2017; Tang et al., 2015). Furthermore, regular mindfulness practice is associated with lower cortisol and inflammatory markers levels, potentially buffering against age-related pathologies such as cardiovascular disease and neurodegeneration (Fountain-Zaragoza and Prakash, 2017; Lutz et al., 2021). Meditation is also linked with structural and functional brain preservation. Chételat et al. (2018) demonstrated that long-term meditation practitioners exhibited higher gray matter volume and glucose metabolism in key brain areas vulnerable to aging, including the prefrontal cortex, cingulate cortex, and insula. These regions are critical for attention, emotional regulation, and self-awareness, and are commonly affected in Alzheimer’s disease. Their findings suggest that meditation may contribute to brain reserve and delay cognitive decline. Moreover, mindfulness-based interventions have been shown to alleviate anxiety, depression, and loneliness, all of which are risk factors for dementia and poor aging outcomes. Meditation enhances telomerase activity, thus potentially promoting longevity. In summary, mindfulness and meditation offer a promising, low-cost approach to foster resilience against psychological stress and support cognitive and emotional health in older adults (Chételat et al., 2018; Fountain-Zaragoza and Prakash, 2017).

#### 4.2.3 Problem-solving and coping skills training

Problem-solving and coping skills training play a significant role in reducing chronic psychological stress and promoting healthy aging by equipping individuals with practical tools to manage stressors effectively. The study by Nakao et al. demonstrated that shogi-assisted cognitive-behavioral therapy (S-CBT) improved problem-solving skills and self-reinforcement among elderly men, particularly those with low subjective wellbeing. The intervention enhanced participants’ ability to manage stress through structured techniques, including distraction activities, behavioral activation, and self-reinforcement, resulting in improved cognitive-behavioral functioning (Nakao et al., 2019). Similarly, Koolhaas et al. (2015) highlighted the potential of problem-solving interventions in workplace settings to enhance sustainable employability among aging workers. Although their study did not show significant improvements in primary outcomes, such as work ability and vitality, it did reveal positive effects on secondary measures, including work attitude, self-efficacy, and skill discretion, suggesting that problem-solving training can foster resilience and adaptive coping strategies. Both studies underscore the importance of tailored interventions that address individual needs and contexts. For instance, Nakao et al. (2019) emphasized the value of integrating leisure activities, such as shogi, to make cognitive-behavioral techniques more engaging, while Koolhaas et al. focused on workplace-based strategies to empower workers. Together, these findings suggest that problem-solving and coping skills training can mitigate stress by promoting adaptive behaviors, enhancing self-efficacy, and improving psychosocial outcomes, thereby supporting healthier aging. However, the effectiveness of such interventions depends on proper implementation, including adequate training for facilitators and active participant engagement (Koolhaas et al., 2015; Nakao et al., 2019). In another study by Richer et al. (2025), a 6-week stress management training (SMT) program for older adults was evaluated, demonstrating significant short-term improvements in problem-solving coping strategies, anxiety reduction, and cortisol regulation. While the intervention effectively enhanced proactive coping and decreased diurnal cortisol levels (AUCg) post-intervention, these benefits were not fully sustained at the 3-month follow-up, except for cortisol reduction. The group-based format likely contributed to success by fostering social support, a key resilience factor. However, high attrition among distressed participants and a sample skewed toward high-end private residences limit generalizability. The findings highlight the potential of brief, psychoneuroendocrinology-informed interventions to mitigate stress in aging populations but underscore the need for booster sessions to prolong effects (Richer et al., 2025).

### 4.3 Medical and therapeutic interventions

#### 4.3.1 Psychotherapy

Psychotherapy plays a crucial role in reducing chronic psychological stress and promoting healthy aging by enhancing mental wellbeing, emotional regulation, and resilience among older adults. As individuals age, they often face physical decline, social isolation, bereavement, and economic hardship, all of which contribute significantly to stress and decreased quality of life. Psychotherapy offers tailored interventions that address these multidimensional stressors. Notably, psychotherapeutic approaches such as person-centered therapy, positive psychology, and CBT have shown efficacy in managing emotional disturbances and promoting psychological wellbeing in the elderly (Bar-Tur, 2021; Bosco et al., 2024; Kim et al., 2021; Kim E. et al., 2023). Kim E. et al. (2023) developed a psychotherapy narration model specifically targeting elderly populations, which combined these therapeutic modalities and demonstrated improved emotional satisfaction across various stress-related scenarios. The integration of narrative techniques enabled personalized, accessible emotional support, particularly beneficial for elderly individuals reluctant or unable to attend in-person sessions. Moreover, geropsychology research emphasizes the role of psychotherapy in cultivating resilience and psychological assets, such as life satisfaction, optimism, and purpose in life, which are factors strongly linked to a lower incidence of chronic diseases and enhanced cognitive and physical functioning in older adults (Kim et al., 2021). Studies further indicate that psychotherapy interventions improve sleep quality, reduce depressive symptoms, and enhance subjective wellbeing, especially when delivered in community settings or via remote formats (Bar-Tur, 2021). Psychotherapeutic support also helps develop coping strategies, improve emotional expression, and foster social connectedness, which are essential for mitigating the effects of loneliness and cognitive decline (Bosco et al., 2024).

#### 4.3.2 Medication

Medications play a nuanced and evolving role in reducing chronic psychological stress and promoting healthy aging (Hilmer et al., 2025). While there is no medication specifically designed to target stress itself (Carroll-Duhigg et al., 2025), pharmacological interventions are widely used to manage stress-related symptoms and comorbid psychiatric conditions such as depression, anxiety, and sleep disturbances (Everly et al., 1981). Antidepressants, especially selective serotonin reuptake inhibitors (SSRIs) and serotonin-norepinephrine reuptake inhibitors (SNRIs), have demonstrated efficacy in mitigating the psychological and physiological manifestations of stress (Correia and Vale, 2024). On the other hand, Anxiolytics, including benzodiazepines and buspirone, are effective for acute relief but present risks of dependency and are therefore restricted to short-term use (Edinoff et al., 2021). Emerging research underscores the substantial interplay between chronic stress, neuroinflammation, and depression, suggesting that pharmacological agents with anti-inflammatory properties may offer novel therapeutic benefits. For instance, recent studies have highlighted that statins, commonly used for cardiovascular risk reduction, exhibit anti-inflammatory effects that can attenuate neuroinflammation and potentially ameliorate depressive symptoms, especially simvastatin, which has shown a moderate effect in clinical trials (Abbasi et al., 2015; Giorgi et al., 2021; Hassamal, 2023; Redlich et al., 2014). Furthermore, modulation of the kynurenine pathway and the inflammatory cascade is increasingly recognized in the context of stress-induced mood disorders, opening avenues for targeted therapeutics targeting cytokines and related pathways (Mariotti, 2015). In the domain of healthy aging, medications originally developed for disease treatment are being studied for their geroprotective properties. Metformin, a long-standing antidiabetic drug, is at the forefront of this movement. Robust epidemiological and clinical evidence indicate that metformin not only reduces the incidence of type 2 diabetes and cardiovascular complications but also positively influences multiple age-related diseases, including cancer, cognitive decline, and all-cause mortality in both diabetic and non-diabetic individuals (Espinoza et al., 2023; Kulkarni et al., 2020; Soukas et al., 2019). Preclinical studies and clinical trials such as Metformin In Longevity Study (MILES) and Targeting Aging with Metformin (TAME) support its capacity to modulate key aging mechanisms, improve metabolic health, and potentially extend health span by activating AMP-activated protein kinase (AMPK) and reducing cellular senescence and systemic inflammation (Espinoza et al., 2023; Mohammed et al., 2021). However, while the literature is promising, conclusive evidence for lifespan extension in healthy humans is still pending, and large-scale longitudinal trials are ongoing to clarify these effects (Keys et al., 2025; Soukas et al., 2019).

Nicotinamide adenine dinucleotide (NAD+) serves as a key redox cofactor and substrate for enzymes, including sirtuins (SIRTs), CD38, and poly ADP-ribose polymerase (PARPs), that are implicated in several biological processes, such as DNA repair, metabolism, and stress resilience. Given that NAD+ levels diminish with age and the decline in NAD+ metabolism aggravates numerous age-related diseases, it is crucial to sustain NAD+ levels for their prevention and treatment (Katayoshi et al., 2023; Yaku et al., 2018). Supplementation with NAD+ precursors such as nicotinamide mononucleotide (NMN) restores the level of NAD+ in animal models, enhancing insulin sensitivity, vascular function, neuroprotection, and physical endurance, and in certain instances prolongs the life span (Das et al., 2019; Picciotto et al., 2016; Mills et al., 2016). Human trials show that a randomized, double blind, placebo-controlled experiment including middle-aged healthy participants shown that 12 weeks of NMN supplementation (250 mg/day) significantly elevated circulating NAD+ metabolism and was well tolerated, with no side effects reported. While arterial stiffness showed a tendency to improve, the alterations did not achieve statistical significance, indicating possible vascular advantages that necessitate additional exploration (Katayoshi et al., 2023) Moreover, in another randomized, multicenter, placebo-controlled study shown that 60 days of NMN supplementation (300–900 mg/day) resulted in dose-dependent elevations in blood NAD+ level, increased physical performance, and enhanced subjective health assessment, all without safety issues. The clinical efficacy peaked at 600 mg/day; however, insulin resistance showed slight variation among the treatment groups (Yi et al., 2023).

Sirtuins (SIRT1-7) are NAD+ dependent deacylases that modulate mitochondrial biogenesis, inflammation, chromatin structure, and metabolic adaptation, playing as key longevity regulators in various organisms. The overexpression of sirtuins or pharmacological activation via NAD+ precursors or sirtuin-activating compounds (STACs) enhances the organ function and resistance to age-related diseases in mice, with some models showing lifespan extension (Bonkowski and Sinclair, 2016). The activity of sitruins depends on NAD+ availability, so combined approaches that elevate NAD+ [e.g., NMN, Nicotinamide riboside (NR)] and directly activate SIRT1 (e.g., resveratrol, synthetic STACs such as SRT2014) are being examined as synergistic geroprotective strategies. However, translation to humans remains preliminary, with ongoing trials primarily targeting metabolic, cardiovascular, and neurodegenerative diseases rather than lifespan (Imai and Guarente, 2014). Rapamycin (sirolimus) inhibits mTORC1, a key kinase responsible for coordinating growth pathways and nutrient sensing, thereby mimicking some effects of dietary restriction and suppressing anabolic signaling associated with aging. In yeast, flies, and mice, chronic or intermittent rapamycin robustly extends median and maximal lifespan, reduces tumor burden, and preserves physiological function (Ehninger et al., 2014; Ivimey-Cook et al., 2025). In vertebrate meta-analysis, rapamycin showed lifespan benefits comparable in magnitude to dietary restriction, reinforcing mTORC1 as a key target of geoscience pharmacology. Nonetheless, in humans, rapamycin and rapalogs remain the primary immunosuppressants, and current off-label longevity use is constrained by concerns over dyslipidemia, mucositis, impaired wound healing, and potential infection risk, necessitating investigation of low-dose or intermittent regimens (Ivimey-Cook et al., 2025; Roark and Iffland, 2025).

Resveratrol, a polyphenol found in grape skin, peanuts, and berries, is the prototypical sirtuin-activating compound (STAC) that has been reported to activate SIRT1 and improve metabolic health in preclinical models. In lower organisms and some rodent studies, resveratrol reduces oxidative stress and inflammation, activates AMPK, and can extend lifespan, particularly under high-fat dietary conditions (Bonkowski and Sinclair, 2016; Zhang et al., 2021). Human trials illustrate modest benefits on insulin sensitivity and some cardiometabolic markers, but its low bioavailability, off-target effects, and heterogenic clinical outcomes have necessitated a strategic shift. Current research is increasingly focused on the development of next-generation STACs engineered with a superior pharmacokinetic profile and enhanced target selectivity (Guarente et al., 2024; Lee et al., 2025).

Ultimately, a pharmacological approach to stress reduction and healthy aging is most effective within a comprehensive strategy that integrates lifestyle modifications, digital interventions, and psychosocial therapies. The literature consistently emphasizes that while medications are valuable adjuncts, they rarely cure stress or prevent aging on their own. Ongoing research into anti-inflammatory and senolytic agents, and personalized regimens tailored to an individual’s comorbidity profile and risk factors, continues to expand the horizons of this interdisciplinary field (Espinoza et al., 2023; Shchaslyvyi et al., 2024; Thanapairoje et al., 2023).

#### 4.3.3 Digital interventions

Artificial intelligence (AI) and telemedicine applications in mental health have demonstrated promising capabilities for stress monitoring and intervention. AI models trained on wearable data have reported high accuracy in stress detection using indicators such as heart rate variability, skin temperature, and galvanic skin response (Dham et al., 2021; Gedam et al., 2025).

More recently, generative AI–based agents have been evaluated for mental health support and intervention. A randomized controlled trial of 210 adults with major mental disorders reported improvements in clinical symptoms after an average of 6 h of engagement with an AI therapy chatbot (Heinz et al., 2025). In parallel, advances in natural language processing (NLP) have strengthened AI’s ability to detect stress and mental disorders by analyzing linguistic features in text and speech (Ibrahim et al., 2025). NLP has also enhanced self-guided intervention, allowing individuals to receive and progress through structured therapeutic content with minimal or no external support, and has shown effectiveness in reducing depressive and anxiety symptoms (Villarreal-Zegarra et al., 2024). In Telemedicine, communication technologies are used to deliver healthcare remotely. Across multiple studies, telemedicine and face-to-face therapy have consistently demonstrated equivalent clinical outcomes for several mental disorders, including depression and anxiety (Chen et al., 2024; Krzyzaniak et al., 2024; Shaker et al., 2023).

AI-driven applications for the prevention and intervention of chronic psychological stress hold promise for expanding access to care in remote areas, enhancing continuity of care, and enabling personalized interventions. Despite encouraging evidence, significant challenges remain in implementing and scaling these applications, including ethical and governance issues related to data privacy, limited empathic responsiveness, algorithmic bias, and limited suitability for crisis situations or complex mental disorders (Lee et al., 2025).

Pichowicz et al. (2025) evaluated the comprehensiveness of responses generated by 29 AI-powered chatbot agents in handling suicidal risk scenarios against predefined criteria (e.g., providing emergency contact information, responding consistently and appropriately). None met the criteria for an adequate response, 15 produced marginal responses and 14 produced inadequate responses. In addition, adherence and sustained engagement remain persistent challenges for digital mental health interventions. Evidence increasingly suggests that both engagement and treatment effects can attenuate over time, highlighting the need for long-term follow-up, interventions designed to support sustained engagement, and the use of hybrid care models when appropriate (Garrido et al., 2019; Jia et al., 2025; Sit et al., 2022; Torous et al., 2020).

### 4.4 Multi-component and community-based approaches

Multi-component and community-based approaches are increasingly recognized as effective strategies for reducing chronic psychological stress and promoting healthy aging, with the literature supporting their potential to address both physiological and psychosocial dimensions of stress while fostering broader wellbeing. Meta-analytic evidence demonstrates that psychological interventions, such as mindfulness-based stress reduction (MBSR), CBT, progressive relaxation, and cognitive restructuring, consistently reduce both subjective and biological markers of stress, including improved immune function and altered cortisol levels in healthy adults (Rogerson et al., 2024; Schakel et al., 2019). However, heterogeneity in intervention components, delivery formats, and engagement levels across studies complicates direct comparisons. It underscores the need for tailored, multimodal programs that integrate cognitive, behavioral, physical, and social elements (Schakel et al., 2019). Randomized controlled trials illustrate the added value of multi-component interventions. For example, a multidisciplinary program integrating workplace-focused psychotherapy and MBSR not only reduced stress symptom levels but also significantly increased return-to-work rates among employees on sick leave due to work-related stress, compared to usual care (Netterstrøm et al., 2013). Similarly, even brief multimodal prevention programs, incorporating health coaching, relaxation, physical activity, and psychoeducation, yielded meaningful reductions in perceived and chronic stress, with sustained improvements in wellbeing and quality of life at follow-up, particularly when refresher or booster sessions were included (Kus et al., 2021; Throner et al., 2023). The success of these programs appears to depend on regular practice and participant engagement, with a positive dose–response relationship between self-practice frequency and intervention outcomes (Schakel et al., 2019). Community-based interventions further extend these benefits by leveraging peer support, social networks, and accessible settings to reinforce sustained behavior change, especially relevant in older populations where social isolation can exacerbate stress and limit access to formal services.

In clinical populations, targeted stress management has improved weight management and mental health in children, suggesting that these approaches may interrupt stress-related metabolic dysregulation and unhealthy behaviors that contribute to accelerated aging and chronic disease (Stavrou et al., 2016). For healthcare workers and other high-stress groups, complex interactive programs that include physical activity, mindfulness, and deep breathing have demonstrated feasibility and preliminary effectiveness in increasing the use of stress management strategies (Wang et al., 2023). While the optimal combination of components remains an active area of research, the current evidence base robustly supports multi-component and community-based interventions as a promising, adaptable framework for both mitigating chronic psychological stress and enhancing the prospects of healthy aging across diverse populations.

## 5 Gaps and future direction

Despite growing evidence linking chronic psychological stress and accelerated aging, several gaps remain in this field that limit our understanding of this complex relationship. One gap is the lack of a standardized framework for quantifying chronic psychological stress. Most studies rely on retrospective self-reported questionnaires to measure stress, which are subjective and susceptible to biases such as social desirability bias and recall bias (Althubaiti, 2016).

Another gap is that most studies examine the impact of chronic psychological stress on one or two hallmarks of aging. Although these studies offer valuable insights, they isolate a single mechanism from a complex, interconnected set of mechanisms that drive the aging process. Importantly, the observed impact of stress on a particular hallmark of aging, such as telomere attrition, may not be a direct effect of psychological stress but rather an indirect consequence of stress-induced alterations in other biological pathways. To our knowledge, no *in vivo* study has comprehensively examined the impact of chronic psychological stress across all hallmarks of aging.

Another key gap is the lack of studies investigating the impact of chronic psychological stress across multiple tissue types. Most existing research, particularly studies on telomere length and epigenetic modifications, uses peripheral blood samples. The findings of these studies may be tissue-specific, and the stress-induced effect may differ across tissues. Animal model studies have shown that psychological stress affects telomere length and epigenetics in a tissue-specific manner (Evans et al., 2021; Kang et al., 2017). Additionally, there is an underrepresentation of interdisciplinary approaches that integrate biological, psychological, and social determinants of health in understanding aging and stress adaptation (Jain and Yadav, 2023).

Most of the presented studies rely on a single biomarker of aging, such as telomere length or DNA methylation. However, using a single biomarker may be insufficient to measure the complexity of the multifaceted aging process. Furthermore, studies investigating the impact of psychological stress on telomere length have reported inconsistent findings (Ämmälä et al., 2020; Khalil et al., 2023; Naudé et al., 2023; Salihu et al., 2016). Although DNA methylation provides a more accurate measure of biological aging, different epigenetic clocks have yielded contradictory results (Joshi et al., 2023; Rampersaud et al., 2022). These limitations highlight the need for an integrated, multi-biomarker approach to more reliably capture the diverse aspects of aging.

In the context of prevention, many studies have short trial durations and small sample sizes, which limit our understanding of the long-term efficacy and generalizability of multi-component and community-based interventions for stress and aging. Many interventions demonstrate promising short-term effects on cognitive and physical health, yet their sustained benefit and optimal combination of components remain unclear, particularly across diverse populations and settings (Fessel et al., 2017; Oh et al., 2021).

Furthermore, there is a notable lack of research prioritizing person-centered approaches, cultural diversity, and integrated care, leading to disparities in how interventions are developed and accessed globally. Access barriers and insufficient adaptation to community-specific needs hamper the scalability of effective programs, especially in low- and middle-income countries, where stigma, provider shortages, and socioeconomic constraints remain unaddressed (Cesari et al., 2024). Addressing these gaps will require more rigorous, long-term trials, greater inclusion of underserved populations, collaboration across disciplines, and policies that promote adaptability and equity in intervention delivery.

Moving forward, promising research directions include transitioning from conventional biological studies that focus on a single molecular layer to multi-omics approaches that integrate genomics, epigenomics, transcriptomics, proteomics, metabolomics, and microbiomics. This direction will provide a deeper understanding of the relationship between psychological stress and aging, as well as the complex interactions among aging hallmarks in response to stress. Furthermore, as AI/ML applications advance in biomedical research, multi-omics datasets hold significant potential for discovering novel biomarkers and intervention targets for stress-related outcomes.

Another direction is to prioritize large-scale, prospective longitudinal studies that integrate biological, psychological, and lifestyle factors. Lifestyle is a crucial confounding variable in the relationship between psychological stress and accelerated aging. Psychological stress not only directly affects aging and increases the risk of aging-related diseases, but also indirectly increases the likelihood of adopting other risk factors. It impacts self-control abilities, decreasing the likelihood of an individual engaging in healthy behaviors, such as exercise and a balanced diet, and increasing the likelihood of engaging in unhealthy behaviors, including smoking, an unbalanced diet, and drug use (Fogelman et al., 2021; Huang et al., 2011; Mouchacca et al., 2013; Yoon et al., 2023). Community-based, multi-component interventions, including stress management training, mindfulness, and cognitive-behavioral approaches, show promise for improving coping and reducing HPA axis activation, but their optimal components, delivery formats, and scalability, particularly in diverse and underserved populations, require further exploration.

## 6 Conclusion

Chronic psychological stress accelerates key biological mechanisms, including mitochondrial dysfunction, telomere shortening, inflammation, DNA damage, cellular senescence, and epigenetic alterations, thereby accelerating the aging process. These changes compromise cellular integrity and increase vulnerability to age-related diseases and functional decline. However, important gaps in our understanding of this relationship remain and need to be addressed in future research. Effective strategies, ranging from lifestyle interventions to psychological and behavioral interventions, as well as medical and therapeutic interventions, have shown promise in mitigating stress-related adverse health outcomes.
